# High-risk populations should be screened for MS: Yes

**DOI:** 10.1177/13524585251353044

**Published:** 2025-07-08

**Authors:** Helen Tremlett

**Affiliations:** Faculty of Medicine (Neurology), University of British Columbia and The Djavad Mowafaghian Centre for Brain Health, UBC Hospital, Vancouver, BC, Canada

## Primum non nocere – first, do no harm

### Screening high-risk populations in clinical practice and research settings

Screening is the process of testing or checking for disease when there are no symptoms.^1^ Screening entails potential benefits – early detection of disease, early treatment with the goal of maximizing health outcomes while minimizing harms. Harms can include unnecessary tests, unnecessary treatments, health anxiety, employment issues, lost work productivity, difficulty with obtaining life insurance and financial costs.^2^ Mammography offers a salient example of a screening test used in clinical practice. When offered to specific groups – females aged 50–69 years – this imaging tool can reduce mortality from breast cancer.^2^ Thus, benefits include early detection and improved treatment outcomes. Harms, however, include false positives, overdiagnosis, unnecessary invasive tests, distress, unwarranted surgery and radiation exposure.^2^

Other screening tools are available for use in research settings. For example, in other neurological diseases, such as Parkinson disease (PD), prodromal symptomatology (markers) combined with traditional risk factors (e.g. sex, age, environmental and lifestyle-related exposures and familial history) are used to identify at-risk individuals. Individuals reaching a certain threshold (probability) of being in the prodromal phase of PD are then being offered the opportunity of enrolment in research studies, including clinical trials.^3^ These trials are testing interventions aimed at secondary prevention; stopping or delaying the development of motor-onset PD.^3^ Importantly, this type of experimental approach has shown to be acceptable to members of the target population.^4,5^ Criteria developed under the auspices of the Movement Disorders Society can identify individuals with prodromal Parkinson’s with high specificity (99%), modest sensitivity (67%) and a positive predictive value of 40% over 3 years.^6^

Why don’t we have a similar tool for MS? Unlike PD, where a prodromal phase has been actively discussed and studied since the 1980s, the concept of a prodromal phase in MS was largely dismissed until relatively recently.^7^ While this places the field of MS at a disadvantage, with key gaps in knowledge remaining,^8^ there are also opportunities to capitalize on the progress made in PD and other neurological diseases. Importantly, MS has notable advantages as a candidate disease for early intervention compared to some other neurological conditions. Specifically, early treatment of MS with a disease-modifying drug (DMD) has demonstrated short-term and some long-term beneficial outcomes (tertiary prevention).^9,10^ Moreover, very early treatment of high-risk individuals – those with RIS – has also shown promise, with two different DMDs (with regulatory approval to treat MS) demonstrating superiority over placebo, at least over the short term.^11,12^ Given these advances, conceptually screening high-risk populations for prodromal MS within a research setting is worthy of active pursuit now. Such an approach would also align with the 2024 ‘Pathways to Cures’ goal of stopping MS (secondary prevention) through early detection and treatment.^13^ RIS is, however, rare, and equity issues surrounding MRI access (required to identify individuals with RIS) is a recognized issue.^14,15^ Developing and testing criteria using low-cost, scalable tools and markers (akin to the approach used in PD) to identify those in the prodromal phase of MS has the potential to target a broader population, beyond those able to readily access an MRI for their non-MS specific complaint or issue.^14,15^ Validated criteria for prodromal MS could enable, for example, high-risk populations to be offered screening and future enrolment in clinical trials aimed at preventing or delaying MS onset (secondary prevention) and subsequent progression of disease. Any intervention offered needs to be low-risk, low-burden, acceptable to potential enrollees with a high likelihood of good adherence and, of course, the potential for long-term beneficial outcomes.^3^ Potential attractive candidate interventions could include lifestyle or dietary modifications, Epstein–Barr virus-targeted vaccines, or repurposed oral therapies with excellent, established safety profiles.^3^

### Which high-risk population might benefit from screening for MS in research settings?

Aside from individuals already diagnosed with RIS, probably the highest known at-risk individuals are those who have a monozygotic twin with MS. With an estimated lifetime risk of developing MS being ≈20%–30%,^16^ a Germany-based group have capitalized on this and have been enrolling discordant twin pairs since 2012.^17^ Intriguing findings to date include, for example, a distinct peripheral immunological signature that differentiated the twin pairs.^17^ Another initiative – the Genes and Environment in MS (GEMS) study group – are, as of April/2025 (nationalmssociety.org/news-and-magazine/news/ms-study-alert-gems) recruiting 5000 individuals living in the United States. Target study participants include first-degree relatives of people with MS, as well as individuals with prior exposure to an anti–tumour necrosis factor-alpha drug – a pharmacological agent associated with increased risk of developing MS.^18^ The overarching goal of the GEMS study is to identify which individuals within these high-risk groups will go on to develop MS, and to elucidate the genetic, environmental, and biological factors that modulate this risk. These are just two examples of ongoing efforts that underscore the potential value of targeted screening for MS in a research setting. Data generated from such studies will be essential to define the optimal screening approach(es), including the most useful predictive tests, biomarkers and tools for early detection in which high-risk groups. Results will take some years before they are available, further highlighting the importance of starting such screening-based studies targeting well-defined high-risk populations now.

To summarize, we should be actively considering and offering screening for MS among high-risk populations as part of rigorously designed, ethically sound, and patient-centred research studies, co-developed with representatives of the target population and those with lived experience of MS. It is imperative to establish whether such approaches confer net benefit or harm, and to identify those most likely to derive benefit. To appropriately weigh individual risks against potential benefits, input from diverse stakeholders, combined with shared decision-making, and comprehensive informed consent are critical. Evidence suggests that treating MS early results in better short- and some longer-term outcomes. Thorough targeted screening of well-defined high-risk groups in research settings – guided by robust ethical safeguards and supported by patient engagement – may offer meaningful long-term benefits to future MS patients and society as a whole.
